# Liquid biopsy identifies actionable dynamic predictors of resistance to Trastuzumab Emtansine (T-DM1) in advanced HER2-positive breast cancer

**DOI:** 10.1186/s12943-021-01438-z

**Published:** 2021-11-29

**Authors:** Matteo Allegretti, Alessandra Fabi, Elena Giordani, Cristiana Ercolani, Paolo Romania, Cecilia Nisticò, Simona Gasparro, Vittoria Barberi, Maria Ciolina, Edoardo Pescarmona, Diana Giannarelli, Gennaro Ciliberto, Francesco Cognetti, Patrizio Giacomini

**Affiliations:** 1 Oncogenomics and Epigenetics, IRCSS Regina Elena National Cancer Institute, Via Elio Chianesi, 53, 00144 Rome, Italy; 2 Medical Oncology 1, IRCSS Regina Elena National Cancer Institute, Rome, Italy; 3 Present Address: Precision Medicine Breast Cancer Unit, Scientific Directorate, Health of Woman and Child Department, Fondazione Policlinico Universitario A. Gemelli IRCCS, Rome, Italy; 4 Pathology, IRCSS Regina Elena National Cancer Institute, Rome, Italy; 5 Specialization School in Oncology, Sapienza University of Rome, Rome, Italy; 6 Radiology and Diagnostic Imaging, IRCSS Regina Elena National Cancer Institute, Rome, Italy; 7 Biostatistical Unit, IRCSS Regina Elena National Cancer Institute, Rome, Italy; 8 Scientific Direction, IRCSS Regina Elena National Cancer Institute, Rome, Italy; 9 Clinical and Molecular Medicine, Sapienza University of Rome, Rome, Italy

**Keywords:** HER2^+^ breast cancer, T-DM1, Liquid biopsy, Circulating tumor DNA, Pharmacological resistance

## Main text

Breast carcinomas of the HER2-positive subtype (HER2 BC) are oncogene addicted, e.g. they rely on a single dominant cancer driver. Pathway hyperactivation is successfully counteracted by a variety of therapeutic agents (small molecules and antibodies) mostly in association with chemotherapy [1, 2]. Recently approved in the adjuvant setting [3], for many years T-DM1 has been standard of care (SoC) in advanced HER2 BC following Trastuzumab/Pertuzumab treatment, although lesser than expected objective responses were observed [4, 5]. Pharmacological resistance to T-DM1 has been associated with several direct or bypass alterations of the HER2 pathway (reviewed in [6]), but most of these were observed in preclinical models only [7–11]. Liquid biopsy (LB) provides instead a unique opportunity to non-invasively capture resistance traits in the clinical setting [12].

### Patients and study design

The LiqBreasTrack cohort study was conducted at the Regina Elena National Cancer Institute from November 2016 to February 2021 to assess tumor molecular alterations occurring in blood under T-DM1 pressure, and recapitulate adaptive tumor evolution in archival tissues (Fig. S1). Eligibility and T-DM1 administration were as per SoC. Demographics and clinical pathological features are presented in Table 1. The study was approved by the competent Ethical Review Board (RS-857/16). Patients signed a written informed consent including the option of re-biopsy. Tumor tissues (*n* = 28) and blood drawings (*n* = 337) were tested by targeted NGS and dPCR (Supplementary Methods and Fig. S2a-b). Progression-free survival (PFS) was calculated between the first T-DM1 administration and progressive disease or last follow-up. Data elaboration was by descriptive statistics and GraphPAD Prism v8.3 (GraphPad Software, CA, USA).

**Table 1:** Demographics and clinical pathological features of LiqBreasTrack-enrolled patients

Characteristics	N (%)
Age, years (range)	56.8 (39.4–83.5)
ECOG Performance Status ≤2	22 (100)
IHC molecular markers	
*Primary tumor tissues*	22
ER+ and/or PgR+	15 (68.2)
ER- and/or PgR-	7 (31.8)
HER2 1+/SISH or FISH+	3 (13.6)
HER2 2+/SISH or FISH+	2 (9.1)
HER2 3+	17 (77.3)
*Metastatic tumor tissues*	9
ER+ and/or PgR+	6 (66.7)
ER- and/or PgR-	3 (33.3)
HER2 1+/SISH or FISH+	1 (11.1)
HER2 2+/SISH or FISH+	3 (33.3)
HER2 3+	5 (55.6)
Previous lines of therapy	
1	14 (63.6)
2	7 (31.8)
3	1 (4.5)
*Pertuzumab as first line treatment*	
Yes	9 (40.9)
No	13 (59.1)
Dominant Metastatic sites	
Liver	3 (13.6)
Lung	3 (13.6)
Bone	5 (22.7)
Soft tissues	7 (31.9)
Brain	4 (18.2)
Number of metastatic sites per patient	
1	8 (36.6)
2	9 (40.9)
≥ 3	5 (22.7)

Previous therapy lines included: Lapatinib plus Capecitabine, Trastuzumab plus Vinorelbine, Trastuzumab plus Carboplatin

*IHC* Immunoistochemistry, *ER* Estrogen receptor, *PgR* Progesterone receptor, *SISH/FISH* Silver in situ hybridization/Fluorescent in situ hybridization

## Results and discussion

### Clinical response to T-DM1

Twenty patients were compliant with the study plan, 2 are still on treatment at the time of writing with no sign of progression, and 2 were lost to follow-up. Partial response (PR), stable disease (SD) and progressive disease (PD) were seen in 12 (60%), 5 (25%) and 3 (15%) evaluable patients, respectively. No complete response was observed.

### Progressive reversal of HER2 amplification in tissues and blood

**Fig. 1:**
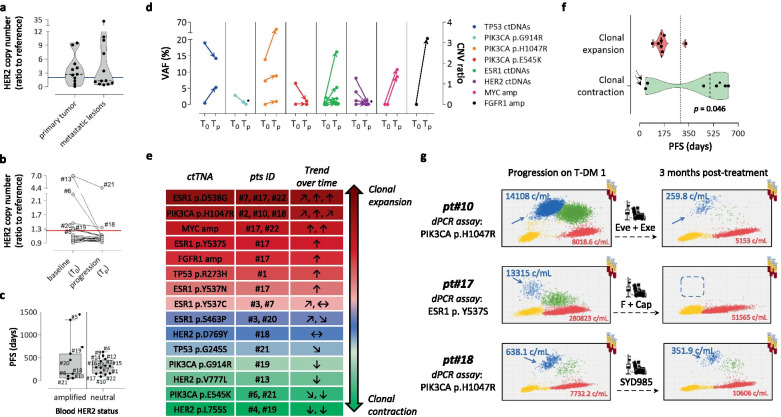
Liquid biopsy identifies actionable dynamic predictors of T-DM1 resistance. **a**, **b** HER2 amplification levels were assessed by dPCR in **a** archival tissues (primary vs metastatic lesions, from left to right), and **b** serial blood drawings (baseline vs progression). Threshold amplification values in tissues (blue line) and blood (red) are shown. **c** Box plot showing patient PFS according to the baseline blood HER2 status. Median PFS and standard deviation are indicated in each box plot. **d** Type, abundance and trends (T_0_ vs T_p_) of circulating alterations. **e** Blood alterations were ranked by trend, consistency and magnitude (slope of the change, straight and slanted arrows). Alterations consistently increasing and decreasing in all patients were color-coded red and green, respectively, whereas inconsistent trends were shown in blue. **f** Violin plot showing PFS of patients with ‘red’ and ‘green’ alterations, respectively. Arrows highlight ‘green’ outliers, e.g. two patients progressing due to brain metastasis with no evidence of increase in blood alterations. The dotted vertical line represents median PFS. **g** Target alterations detected during T-DM1 administration (left panels) were monitored at the time of progression and during subsequent off-label treatments, as indicated. Red, blue and green dots identify WT, mutated and double dPCR positives. Copies/ml of WT (red + green) and mutated (blue + green) DNAs in blood plasma are indicated. *: patient still on T-DM1 treatment. PFS: progression-free survival. VAF: variant allele frequency. Cap: Capecitabine. Exe: Exemestane. Eve: Everolimus. F: Fulvestrant

Tissues and plasma from the LiqBreasTrack study were tested by a dPCR assay shown by others to quantitatively detect HER2 amplification [13, 14]. Due to normal DNA present in blood and in tissues with an abundant stromal component, absolute copy numbers are underestimated by the assay [14, 15]. Nevertheless, dPCR was accurate and quantitative also in our hands, as shown by its remarkable concordance with NGS (Fig. S2c-d). Testing all samples under identical conditions clearly documented progressive HER2 counter-selection. HER2 amplification was detected in 7/11 (64%) primary tumors but only 5/12 (42%) metastatic lesions collected during previous anti-HER2 treatments, and in 7/20 (35%) blood drawings collected before T-DM1 treatment, but only 2/20 (10% overall) blood drawings at progression (Fig. 1a-b). HER2 counterselection in blood was confirmed in 3/4 matched (from the same patient) tumor re-biopsies at progression, the only exception being a HER2-positive brain metastasis developing against a HER2-neutral blood background (pt#5; Fig. S3). Interestingly, median PFS did not significantly differ depending on the HER2 blood status (amplified vs neutral) at baseline (Fig. 1c). Perhaps, like Trastuzumab Deruxtecan [16] T-DM1 remains active on tumors with attenuated HER2 signaling, e.g. a HER2-neutral, but druggable, status spans a much larger patient cohort and a much wider time window than appreciated so far.

### Remodeling in oncogenic dependencies

Breast cancer alterations other than HER2 amplification were identified and sorted out in 3 steps. First, orthogonal testing with targeted NGS and alteration-specific dPCR assays of 28 tumor tissues (patient *n* = 14) and 337 plasma samples (patient *n* = 20) concordantly detected 150 and 27 mutational hits, respectively. Second, dPCR testing detected 3 of the above hits in genomic DNAs from the peripheral blood mononuclear cells (PBMCs) of 2 distinct patients, demonstrating the occasional origin of some alterations from clonal haematopoiesis (Fig. S4). Filtering these 3 hits out left 147 and 24 hits in tissues and plasma respectively, all deemed to represent genuine breast cancer alterations. Third, counting each alteration once (several hits recurred in different samples and patients) yielded a total of 136 and 15 unique tumor variants. A synopsis of patients, clinical-biological features, and a list of genes with detectable alterations is displayed in Fig. S5a-b. Interestingly, 14 of the 15 unique tumor variants seen in plasma were detected in a subset of 12 patients with at least one available, matched tissue sample, making it possible to calculate that only 7 variants were shared between tissue and blood in this representative subset, whereas the remaining 7 were observed in blood only (Fig. S5c). Altogether, HER2 neutralization and the appearance of new variants in blood suggest an extensive remodeling in oncogenic dependencies that would have been missed by tissue-only bulk sequencing.

### LB dynamics hint at several distinct clonal selection mechanisms

Since blood was drawn every 21 days, on the occasion of each T-DM1 administration, detailed clonal trajectories could be assessed. Alterations undergoing at least two consecutive > 1.5-fold increases in their VAFs were assumed to mark clonal expansion. Depending on whether immediately evident or delayed (e.g. since the first blood drawing or afterwards), clonal expansions were consistent with primary and adaptive pharmacological resistance, respectively (Fig. S6a-b). In contrast, clonal contractions (defined as a reduction by > 50%) were invariably steep, e.g. they occurred abruptly, typically after a single T-DM1 administration (Fig. S6c). For instance, HER2 and some PIK3CA mutations known to confer resistance to previous anti-HER2 treatments [17, 18] were irreversibly wiped off within weeks despite they had been selected during years of previous therapies, as documented in archival tumor tissues (Fig. S5a). Overall, swift clonal suppression provides a rationale for innovative pulse dosing/de-escalation schedules. In selected patients, these may elicit response with minimal treatment-associated toxicity.

### LB anticipates progression at extracranial locations

The LiqBreasTrack design takes advantage of a narrow (Fig. S1) but sensitive (Fig. S2a) targeted NGS panel to detect a few major cancer drivers. Therefore, it was not expected to detect circulating alterations in most patients. Accordingly, 10/18 (54%) patients who were monitored until progression did not display alterations or quantitative changes in their levels (6 and 4 patients, respectively). However, and interestingly, all 3 patients who progressed exclusively due to cerebral metastases (Fig. S7) were included in this non-informative, LB-negative subset. This is not surprising since brain involvement is best monitored through the cerebrospinal fluid [19]. In the remaining 8 patients (44%) progression as per medical imaging was anticipated by 2.6 (range 0.7-4.6) months on average. Although shorter than in other settings, this anticipation may be clinically useful since the expected median PFS during T-DM1 treatment is about 6.4 months (range 4.8-7.7 months) in real life studies [4].

### LB identifies positive and negative PFS predictors

Most likely due to the limited case accrual, none of the mutated genes and variants seen in either tissue or blood at baseline significantly correlated with PFS (not shown). We then hypothesized that dynamic, LB-informed criteria might better identify variants associated with different outcomes. Then, the VAFs of tumor variants were graphed (baseline vs progression) as in Fig. 1d. Three distinct trends were evident: some variants (e.g. PIK3CA p.H1047R) consistently increased in all the patients in whom they were observed, others (e.g. HER2 mutations) consistently decreased, and others yet (e.g. TP53) were inconsistent, e.g. they displayed increases in some patients and decreases in others. Alterations were then sorted by trend, color-coded (red for consistent increases, green for consistent decreases, and blue for inconsistent changes), and ranked for the number of patients in whom they had been observed. Additional ranking for magnitude of the observed change resulted in the pseudocolor distribution shown in Fig. 1e. Consistent trends were accepted, whereas inconsistent observations/alterations were rejected because uninformative with respect to clonal outcome. Patients with at least one red alteration, even in presence of green co-mutations, were assumed to carry a dominant negative predictor, whereas exclusive presence of a green alteration was hypothesized to be a positive predictor. Although larger numbers are needed to draw firm conclusions, this dynamic classification identified two groups of patients with significantly (*p* < 0.05) different PFS (Fig. 1f). Interestingly, most PFS values of patients bearing negative predictors clustered far below the median, suggesting that negative predictors are particularly robust and possibly coincide with drivers of clinical resistance to T-DM1. Positive and negative predictors were confirmed by preliminary analysis including 12 additional patients from a larger ongoing multicenter study (not shown).

### Circulating predictors are actionable

Interrogation of the OncoKB knowledge base [20] revealed that 20/24 (83%) blood variants in 13/18 (72%) of the patients progressing on T-DM1 were actionable, mostly at level 3A, e.g. in indication for non-HER2 advanced BC (Table S1). This suggests bypass of the HER2 blockade through forced, and systematic, molecular subtype switch. Some alterations were actionable (e.g. HER2 mutations and lapatinib), but treatment was not considered because drugs had already been used in previous therapy cycles, e.g. these patients were assumed to carry refractory clonal (re)-expansions. Three patients with ESR1 and PI3KCA mutations were prioritized, and either referred to our intramural Molecular Tumor Board, or enrolled in clinical trials for off-label treatment with Fulvestrant, Everolimus plus Exemestane, and SYD987, under a strict LB monitoring scheme. All patients achieved PR lasting 7.2 to 8.6 months, and CT scans were mirrored by blood clearance of the target alteration selected during T-DM1 treatment (Fig. 1g). Thus, at least in these cases, LB identified circulating drivers, and not passengers, of T-DM1 escape. It remains to be determined whether and which alterations listed in Table S1, if any, are actionable in the post-T-DM1 setting.

## Conclusions

In summary, LB identifies drivers/predictors (at extracranial sites only) of T-DM1 escape as they gradually replace HER2, suggesting systematic molecular subtype switch. Predictors of progression may be cryptic (blood-only), and include actionable ESR1 and PIK3CA mutations as well as MYC and FGFR1 amplifications. In contrast, other PIK3CA mutations and all tested HER2 mutations are wiped off by T-DM1 in weeks and may be associated with a more durable T-DM1 response.

## Supplementary Information

**Additional file 1: Fig. S1.** LiqBreasTrack study design. Retrospective (left) and prospective (middle-right) testing of archival tumor tissues and serial blood drawings. Targeted NGS was carried out before the first T-DM1 administration, on the occasion of revaluation by medical imaging, and at progression (*). dPCR with mutation-specific assays was performed on all blood drawings. Re-biopsy was occasionally assessed for confirmatory purposes. FFPE: formalin fixed-paraffin embedded.

**Additional file 2: Fig. S2.** Testing accuracy and correlation statistics. (a) Limit of detection of blood NGS analysis performed in each patient. Values are automatically calculated by the Ion Reporter software v 5.16 as median LOD of all generated amplicons. (b) NGS sequencing depth on archival tissue (left and middle panels) and blood samples (right). Median values are indicated by the dotted line. (c-d) HER2 copy numbers estimated by NGS and dPCR in each plasma sample, and linear regression. (e-f) Linear regression of the abundance (VAF; variant allele frequency) of tumor alterations estimated by NGS and dPCR in tumor tissues and blood, as indicated. Frequency, confidence intervals (grey areas around best fit curve), *p* values and goodness of fit (R) are shown. LOD: limit of detection.

**Additional file 3: Fig. S3.** HER2 amplification in tumor tissue re-biopsies at progression. HER2 amplification was assessed in the last tumor tissue available before T-DM1 administration vs a tumor re-biopsy collected at disease progression from the same patients (*n* = 4). The cut-off value for HER2 amplification (blue line) is shown.

**Additional file 4: Fig. S4.** Clonal hematopoiesis. (a) dPCR testing of DNAs from tumor tissues and PBMCs from the two patients (out of 22) in whom clonal hematopoiesis (3 circulating TP53 mutations) was detected. (b) ‘Zigzagging’ trajectories (no progressive trend for either increase or decrease discernible) of the same alterations in serial blood drawings. Red, blue and green dots: wild-type allele, mutated allele, and double-positive dPCR spots, respectively. NTC: no template control. PBMCs: peripheral blood mononuclear cells. VAF: variant allele frequency.

**Additional file 5: Fig. S5.** Genomic and clinical-biological profiling of tumor tissues and blood. Somatic mutations in (a) tumor tissues and (b) blood samples were arranged by patient number. Top graph: numbers of somatic mutations per patient. Top four rows: best response to T-DM1, and biological characterization of each tumor. All other rows: oncoprint of genomic alterations. Right side of oncoprint: numbers of mutations per gene. (c) Venn diagram: tumor mutational hits in tissue, blood and their intersection. SNVs: single nucleotide polymorphisms.

**Additional file 6: Fig. S6.** ctTNA trajectories during T-DM1 treatment. Representative results of LB with mutation-specific dPCR assays (lines) and ultra-deep NGS (selected time points; bars) in serial blood drawings. Trajectories are consistent with primary (a) and acquired (b) resistance, or response (c) to T-DM1. Shaded areas highlight outcome anticipation by LB (lead time), e.g. the time elapsed from progression (or response) assessed by LB, to progression (or response) assessed by clinical imaging. VAF: variant allele frequency.

**Additional file 7: Fig. S7.** Mutations from brain metastases are undetectable in blood. ETV6 and GATA3 mutations were assessed by dPCR in a brain metastasis surgically removed from pt.#5, and in blood obtained right before surgery. Red, blue and green dots identify WT, mutated and double dPCR positives. Copies per ml of the wild-type allele in plasma are noted. VAF: variant allele frequency.

**Additional file 8: Table S1.** Actionable level of circulating ctTNAs. * OncoKB highest level of evidence in advanced breast cancer.

**Additional file 9.**

## Data Availability

Data supporting the conclusions of this article are available on the IRCCS Regina Elena National Cancer Institute website (www.ifo.it) upon request.

## Abbreviations

ctTNAs: Circulating total tumor nucleic acids
dPCR: Digital PCR
HER2 BC: Breast carcinomas of the HER2-positive subtype
LB: Liquid biopsy
NGS: Next generation sequencing
PBMCs: Peripheral blood mononuclear cells
PD: Progressive disease
PFS: Progression-free survival
PR: Partial response
SD: Stable disease
SoC: Standard of care
T-DM1: Trastuzumab-emtansine
