# Supplementary material for: Liquid biopsy identifies actionable dynamic predictors of resistance to Trastuzumab Emtansine (T-DM1) in advanced HER2-positive breast cancer
Source: Mol Cancer. 2021 Nov 29;20:151. doi: 10.1186/s12943-021-01438-z (PMC8628389; doi:10.1186/s12943-021-01438-z)
Supplement: Supplementary file 1 — Additional file 1: Fig. S1. LiqBreasTrack study design. Retrospective (left) and prospective (middle-right) testing of archival tumor tissues and serial blood drawings. Targeted NGS was carried out before the first T-DM1 administration, on the occasion of revaluation by medical imaging, and at progression (*). dPCR with mutation-specific assays was performed on all blood drawings. Re-biopsy was occasionally assessed for confirmatory purposes. FFPE: formalin fixed-paraffin embedded. [file 12943_2021_1438_MOESM1_ESM.pptx]

## Slide 1
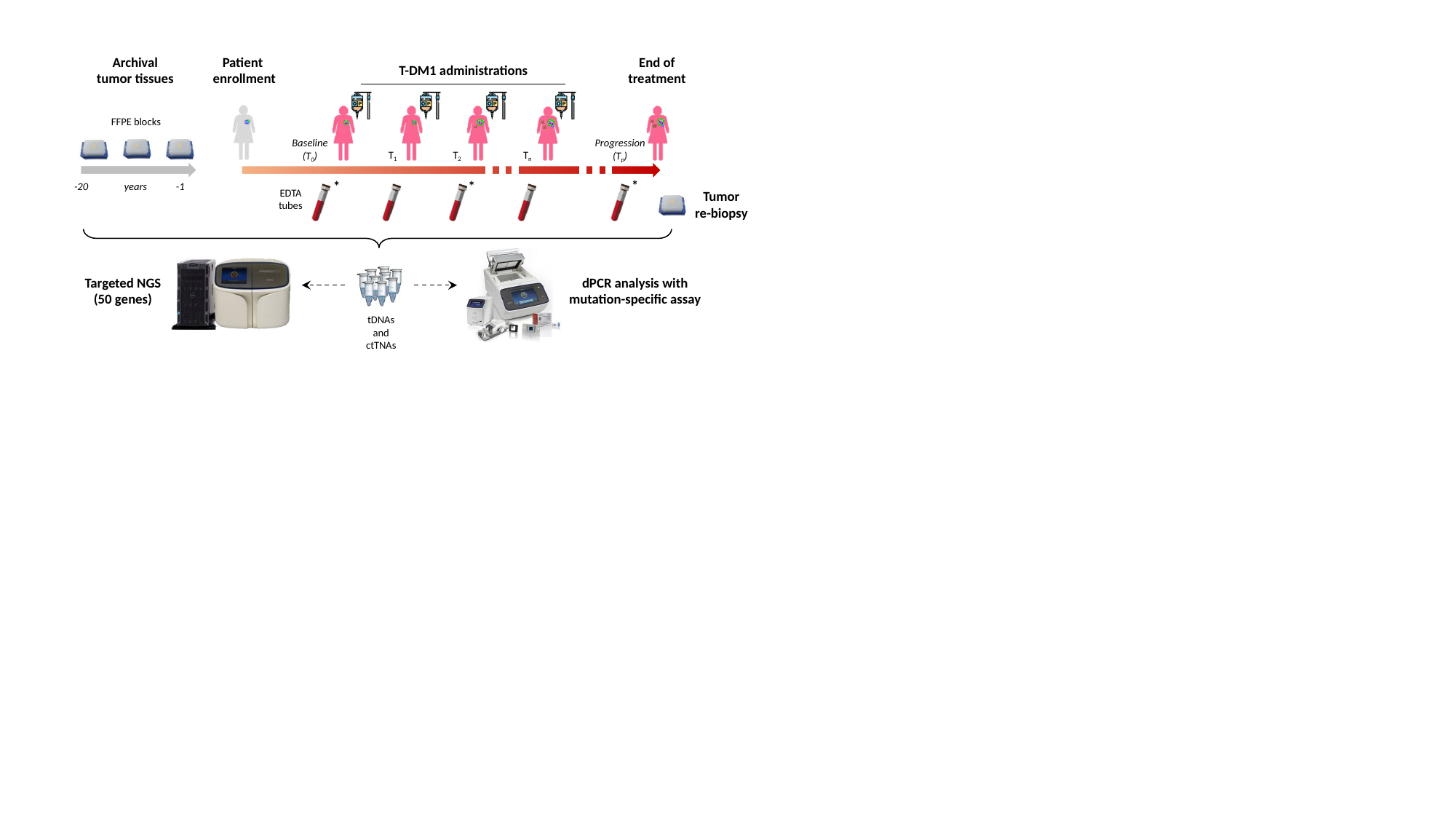

Archival
tumor tissues
Patient
 enrollment
End of treatment
T-DM1 administrations
FFPE blocks
Baseline
(T0)
Progression
(Tp)
T1
T2
Tn
*
*
*
-20
years
-1
EDTA tubes
Tumor re-biopsy
Targeted NGS
(50 genes)
dPCR analysis with mutation-specific assay
tDNAs
and ctTNAs
