# Supplementary material for: Liquid biopsy identifies actionable dynamic predictors of resistance to Trastuzumab Emtansine (T-DM1) in advanced HER2-positive breast cancer
Source: Mol Cancer. 2021 Nov 29;20:151. doi: 10.1186/s12943-021-01438-z (PMC8628389; doi:10.1186/s12943-021-01438-z)
Supplement: Supplementary file 2 — Additional file 2: Fig. S2. Testing accuracy and correlation statistics. (a) Limit of detection of blood NGS analysis performed in each patient. Values are automatically calculated by the Ion Reporter software v 5.16 as median LOD of all generated amplicons. (b) NGS sequencing depth on archival tissue (left and middle panels) and blood samples (right). Median values are indicated by the dotted line. (c-d) HER2 copy numbers estimated by NGS and dPCR in each plasma sample, and linear regression. (e-f) Linear regression of the abundance (VAF; variant allele frequency) of tumor alterations estimated by NGS and dPCR in tumor tissues and blood, as indicated. Frequency, confidence intervals (grey areas around best fit curve), p values and goodness of fit (R) are shown. LOD: limit of detection. [file 12943_2021_1438_MOESM2_ESM.pptx]

## Slide 1
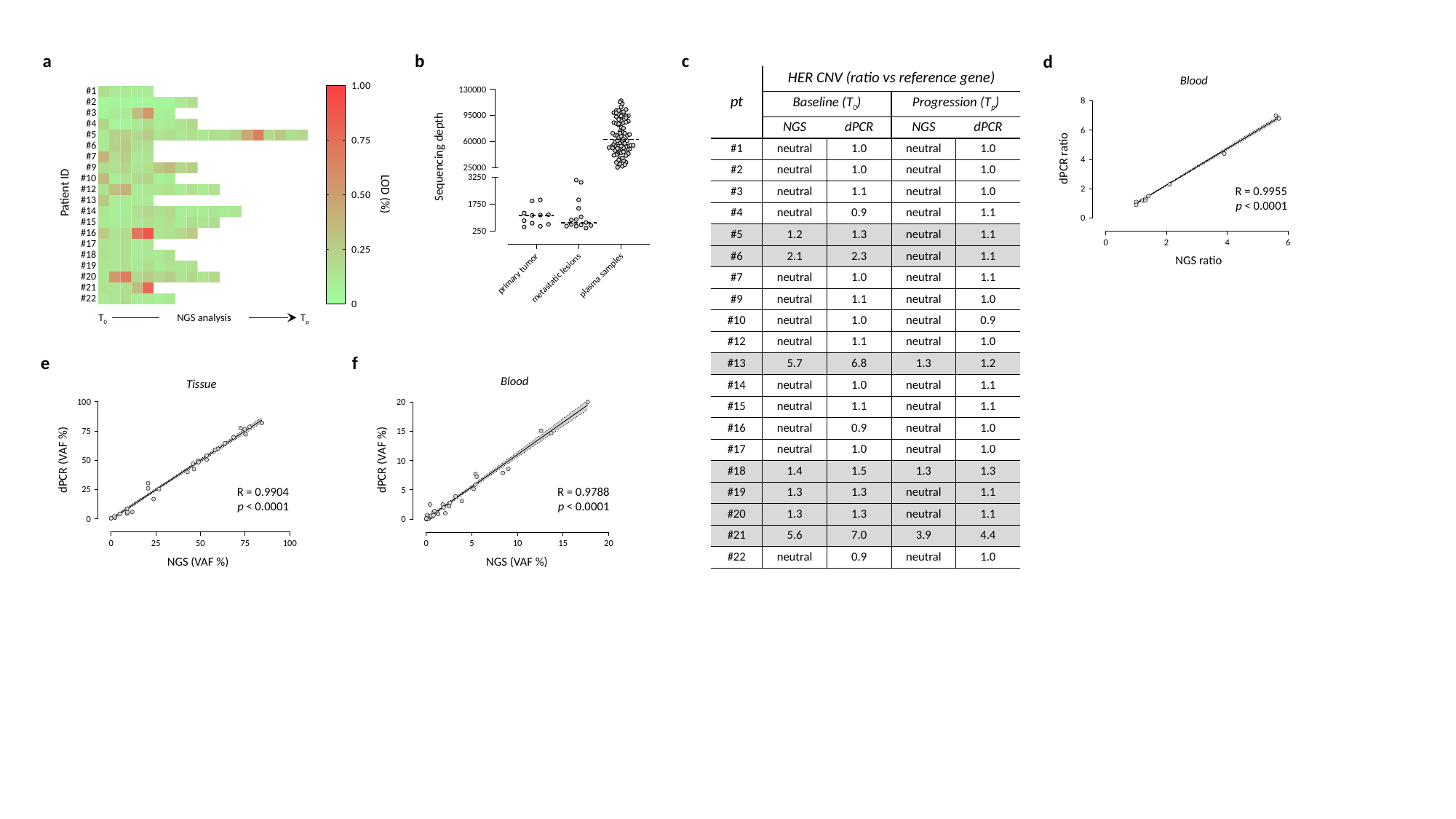

c
b
a
d
| pt | HER CNV (ratio vs reference gene) | | | |
| --- | --- | --- | --- | --- |
| | Baseline (T0) | | Progression (Tp) | |
| | NGS | dPCR | NGS | dPCR |
| #1 | neutral | 1.0 | neutral | 1.0 |
| #2 | neutral | 1.0 | neutral | 1.0 |
| #3 | neutral | 1.1 | neutral | 1.0 |
| #4 | neutral | 0.9 | neutral | 1.1 |
| #5 | 1.2 | 1.3 | neutral | 1.1 |
| #6 | 2.1 | 2.3 | neutral | 1.1 |
| #7 | neutral | 1.0 | neutral | 1.1 |
| #9 | neutral | 1.1 | neutral | 1.0 |
| #10 | neutral | 1.0 | neutral | 0.9 |
| #12 | neutral | 1.1 | neutral | 1.0 |
| #13 | 5.7 | 6.8 | 1.3 | 1.2 |
| #14 | neutral | 1.0 | neutral | 1.1 |
| #15 | neutral | 1.1 | neutral | 1.1 |
| #16 | neutral | 0.9 | neutral | 1.0 |
| #17 | neutral | 1.0 | neutral | 1.0 |
| #18 | 1.4 | 1.5 | 1.3 | 1.3 |
| #19 | 1.3 | 1.3 | neutral | 1.1 |
| #20 | 1.3 | 1.3 | neutral | 1.1 |
| #21 | 5.6 | 7.0 | 3.9 | 4.4 |
| #22 | neutral | 0.9 | neutral | 1.0 |
Blood
dPCR ratio
NGS ratio
Sequencing depth
R = 0.9955
p < 0.0001
Patient ID
LOD (%)
T0
Tp
NGS analysis
e
f
Blood
Tissue
dPCR (VAF %)
NGS (VAF %)
dPCR (VAF %)
R = 0.9904
p < 0.0001
R = 0.9788
p < 0.0001
NGS (VAF %)
