# Supplementary figures and images for: Liquid biopsy identifies actionable dynamic predictors of resistance to Trastuzumab Emtansine (T-DM1) in advanced HER2-positive breast cancer
Source: Mol Cancer. 2021 Nov 29;20:151. doi: 10.1186/s12943-021-01438-z (PMC8628389; doi:10.1186/s12943-021-01438-z)

## Slide 1
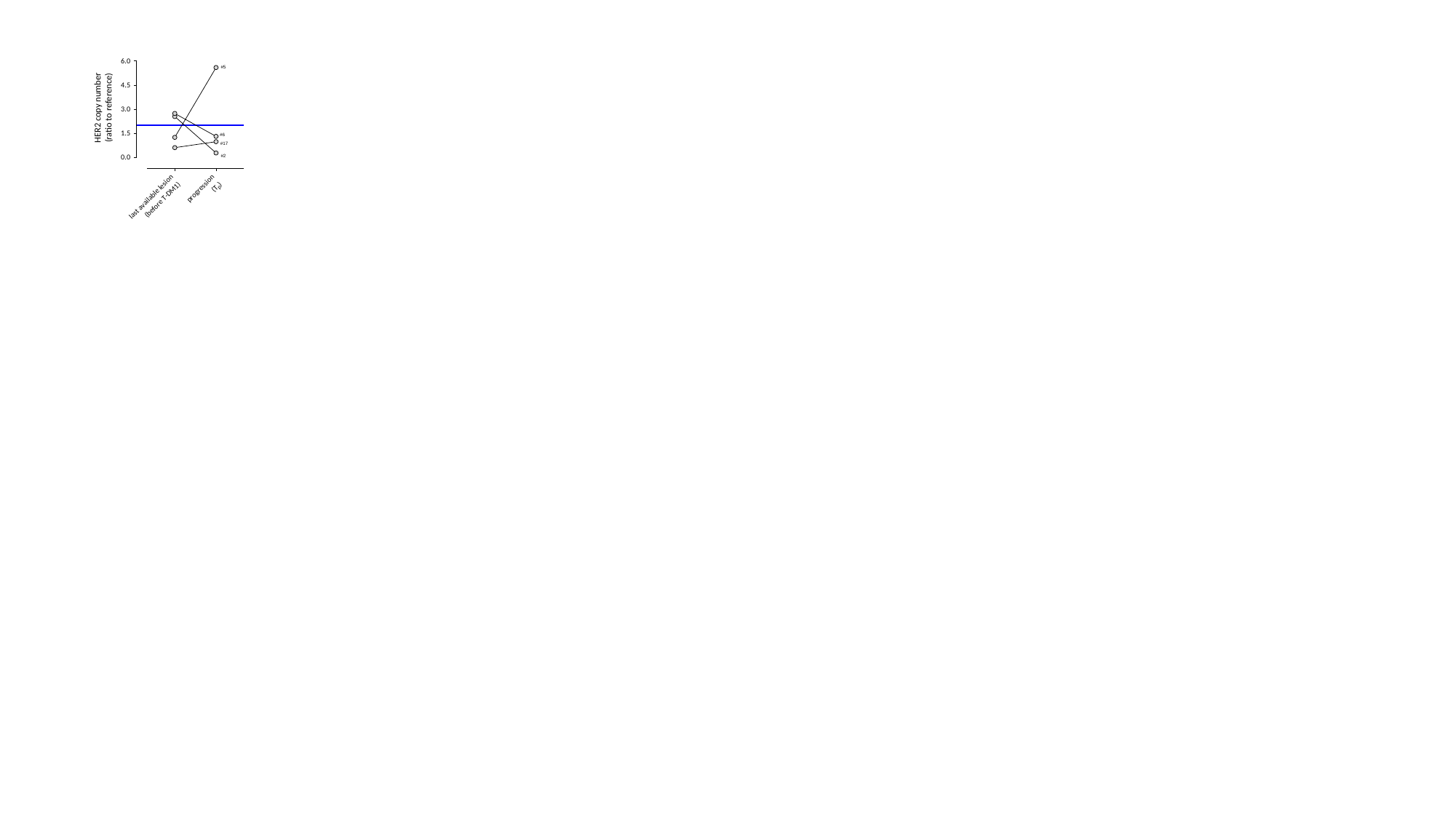

HER2 copy number
(ratio to reference)

Supplement: Supplementary file 3 — Additional file 3: Fig. S3. HER2 amplification in tumor tissue re-biopsies at progression. HER2 amplification was assessed in the last tumor tissue available before T-DM1 administration vs a tumor re-biopsy collected at disease progression from the same patients (n = 4). The cut-off value for HER2 amplification (blue line) is shown. [file 12943_2021_1438_MOESM3_ESM.pptx]
