# Supplementary material for: Liquid biopsy identifies actionable dynamic predictors of resistance to Trastuzumab Emtansine (T-DM1) in advanced HER2-positive breast cancer
Source: Mol Cancer. 2021 Nov 29;20:151. doi: 10.1186/s12943-021-01438-z (PMC8628389; doi:10.1186/s12943-021-01438-z)
Supplement: Supplementary file 4 — Additional file 4: Fig. S4. Clonal hematopoiesis. (a) dPCR testing of DNAs from tumor tissues and PBMCs from the two patients (out of 22) in whom clonal hematopoiesis (3 circulating TP53 mutations) was detected. (b) ‘Zigzagging’ trajectories (no progressive trend for either increase or decrease discernible) of the same alterations in serial blood drawings. Red, blue and green dots: wild-type allele, mutated allele, and double-positive dPCR spots, respectively. NTC: no template control. PBMCs: peripheral blood mononuclear cells. VAF: variant allele frequency. [file 12943_2021_1438_MOESM4_ESM.pptx]

## Slide 1
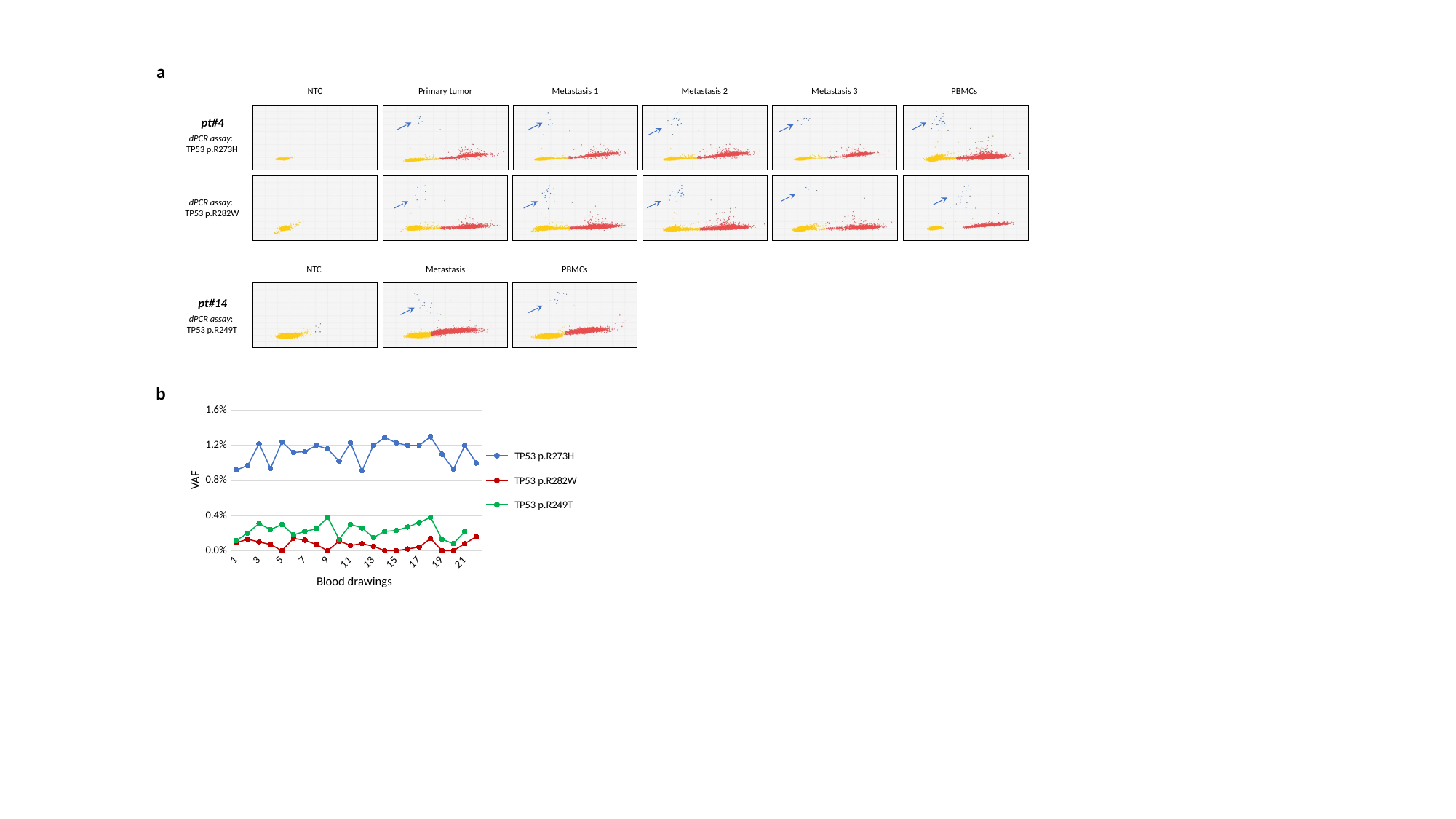

a
Metastasis 3
PBMCs
NTC
Primary tumor
Metastasis 1
Metastasis 2
pt#4
dPCR assay:
TP53 p.R273H
dPCR assay:
TP53 p.R282W
NTC
Metastasis
PBMCs
pt#14
dPCR assay:
TP53 p.R249T
b
[unsupported chart]
TP53 p.R273H
TP53 p.R282W
TP53 p.R249T
VAF
Blood drawings
