# Supplementary material for: Liquid biopsy identifies actionable dynamic predictors of resistance to Trastuzumab Emtansine (T-DM1) in advanced HER2-positive breast cancer
Source: Mol Cancer. 2021 Nov 29;20:151. doi: 10.1186/s12943-021-01438-z (PMC8628389; doi:10.1186/s12943-021-01438-z)
Supplement: Supplementary file 5 — Additional file 5: Fig. S5. Genomic and clinical-biological profiling of tumor tissues and blood. Somatic mutations in (a) tumor tissues and (b) blood samples were arranged by patient number. Top graph: numbers of somatic mutations per patient. Top four rows: best response to T-DM1, and biological characterization of each tumor. All other rows: oncoprint of genomic alterations. Right side of oncoprint: numbers of mutations per gene. (c) Venn diagram: tumor mutational hits in tissue, blood and their intersection. SNVs: single nucleotide polymorphisms. [file 12943_2021_1438_MOESM5_ESM.pptx]

## Slide 1
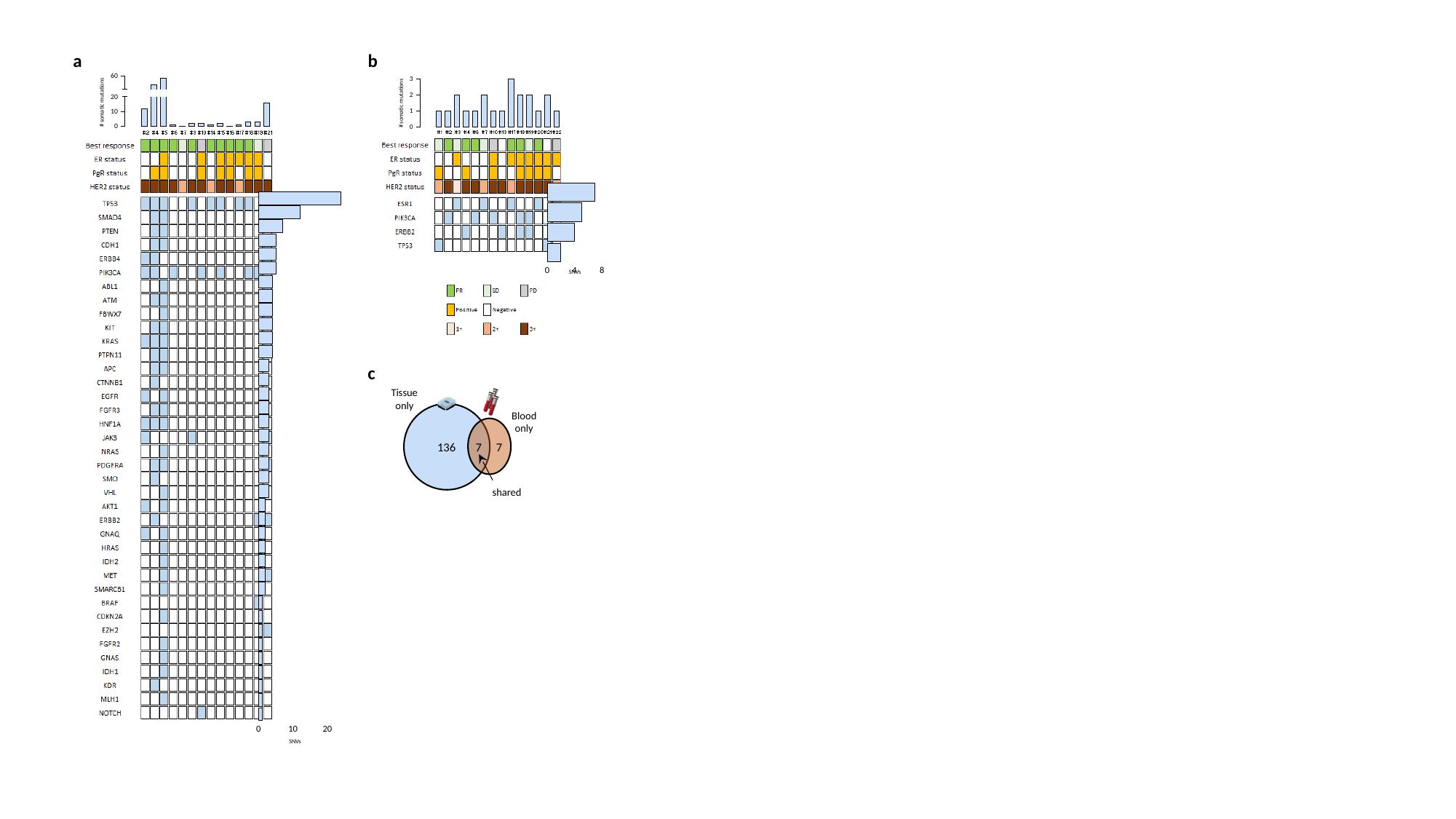

a
b
# somatic mutations
# somatic mutations
### Chart
| Category | |
|---|---|
| BRAF | 1.0 |
| CDKN2A | 1.0 |
| EZH2 | 1.0 |
| FGFR2 | 1.0 |
| GNAS | 1.0 |
| IDH1 | 1.0 |
| KDR | 1.0 |
| MLH1 | 1.0 |
| NOTCH | 1.0 |
| AKT1 | 2.0 |
| ERBB2 | 2.0 |
| GNAQ | 2.0 |
| HRAS | 2.0 |
| IDH2 | 2.0 |
| MET | 2.0 |
| SMARCB1 | 2.0 |
| APC | 3.0 |
| CTNNB1 | 3.0 |
| EGFR | 3.0 |
| FGFR3 | 3.0 |
| HNF1A | 3.0 |
| JAK3 | 3.0 |
| NRAS | 3.0 |
| PDGFRA | 3.0 |
| SMO | 3.0 |
| VHL | 3.0 |
| ABL1 | 4.0 |
| ATM | 4.0 |
| FBWX7 | 4.0 |
| KIT | 4.0 |
| KRAS | 4.0 |
| PTPN11 | 4.0 |
| CDH1 | 5.0 |
| ERBB4 | 5.0 |
| PIK3CA | 5.0 |
| PTEN | 7.0 |
| SMAD4 | 12.0 |
| TP53 | 24.0 |
### Chart
| Category | |
|---|---|
| TP53 | 2.0 |
| ERBB2 | 4.0 |
| PIK3CA | 5.0 |
| ESR1 | 7.0 |SNVs
c
Tissue
only
Blood
only
136
7
7
shared
SNVs
