# Supplementary material for: Liquid biopsy identifies actionable dynamic predictors of resistance to Trastuzumab Emtansine (T-DM1) in advanced HER2-positive breast cancer
Source: Mol Cancer. 2021 Nov 29;20:151. doi: 10.1186/s12943-021-01438-z (PMC8628389; doi:10.1186/s12943-021-01438-z)
Supplement: Supplementary file 6 — Additional file 6: Fig. S6. ctTNA trajectories during T-DM1 treatment. Representative results of LB with mutation-specific dPCR assays (lines) and ultra-deep NGS (selected time points; bars) in serial blood drawings. Trajectories are consistent with primary (a) and acquired (b) resistance, or response (c) to T-DM1. Shaded areas highlight outcome anticipation by LB (lead time), e.g. the time elapsed from progression (or response) assessed by LB, to progression (or response) assessed by clinical imaging. VAF: variant allele frequency. [file 12943_2021_1438_MOESM6_ESM.pptx]

## Slide 1
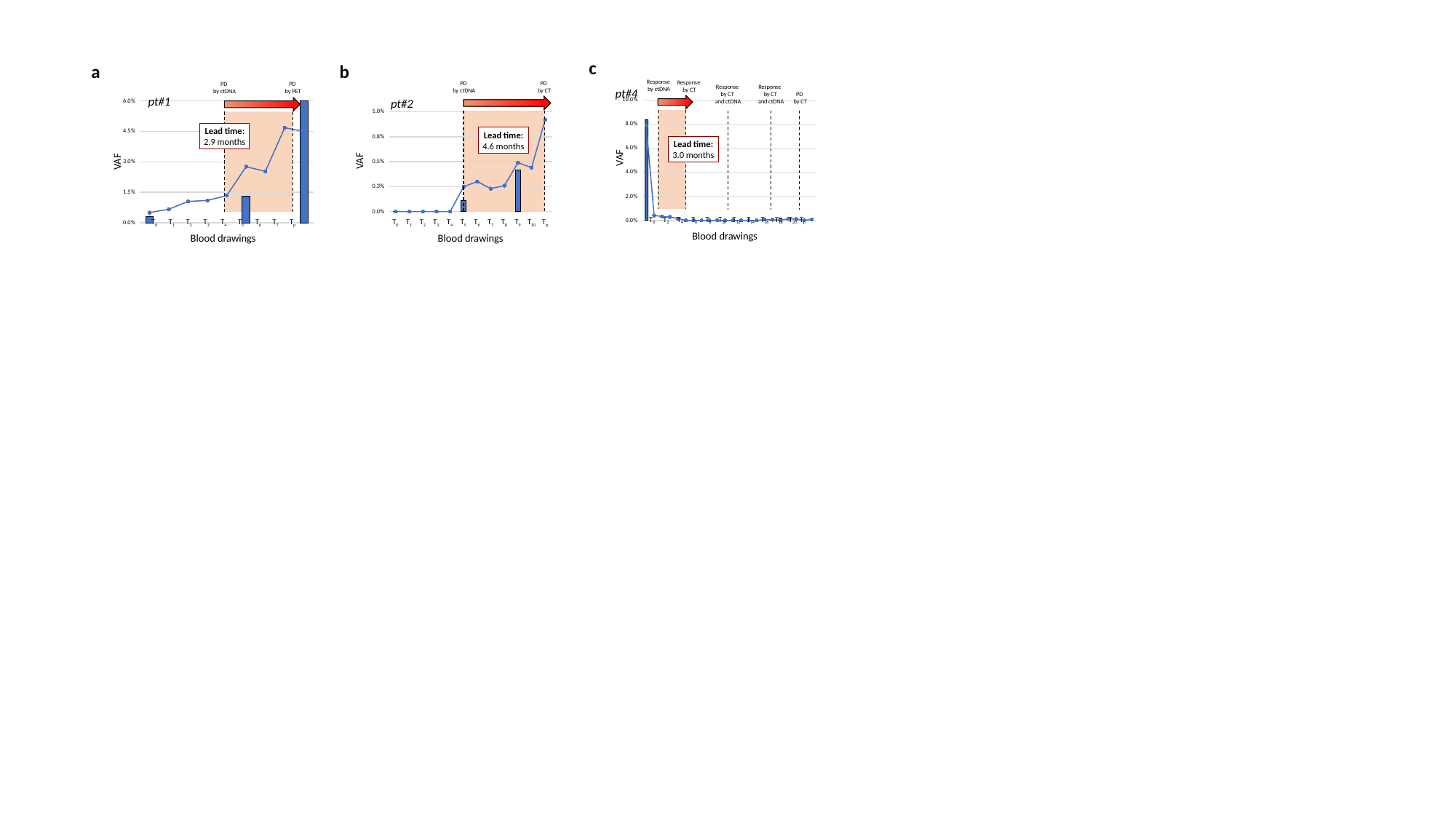

c
a
b
Response
by ctDNA
Response
by CT
PD
by ctDNA
PD
by CT
PD
by ctDNA
PD
by PET
Response
by CT
and ctDNA
Response
by CT
 and ctDNA
pt#4
PD
by CT
pt#1
pt#2
### Chart
| Category | ERBB2 p.L755S | ERBB2 p.L755S |
|---|---|---|
### Chart
| Category | TP53 p.R273H | TP53 p.R273H |
|---|---|---|
### Chart
| Category | PIK3CA p.H1047R | PIK3CA p.H1047R |
|---|---|---|
Lead time:
2.9 months
Lead time:
4.6 months
Lead time:
3.0 months
VAF
VAF
VAF
T0
T2
T4
T6
T8
T10
T12
T14
T16
T18
T20
Tp
T0
T1
T2
T3
T4
T5
T6
T7
Tp
T0
T1
T2
T3
T4
T5
T6
T7
T8
T9
T10
Tp
Blood drawings
Blood drawings
Blood drawings
