# Supplementary material for: Liquid biopsy identifies actionable dynamic predictors of resistance to Trastuzumab Emtansine (T-DM1) in advanced HER2-positive breast cancer
Source: Mol Cancer. 2021 Nov 29;20:151. doi: 10.1186/s12943-021-01438-z (PMC8628389; doi:10.1186/s12943-021-01438-z)
Supplement: Supplementary file 7 — Additional file 7: Fig. S7. Mutations from brain metastases are undetectable in blood. ETV6 and GATA3 mutations were assessed by dPCR in a brain metastasis surgically removed from pt.#5, and in blood obtained right before surgery. Red, blue and green dots identify WT, mutated and double dPCR positives. Copies per ml of the wild-type allele in plasma are noted. VAF: variant allele frequency. [file 12943_2021_1438_MOESM7_ESM.pptx]

## Slide 1
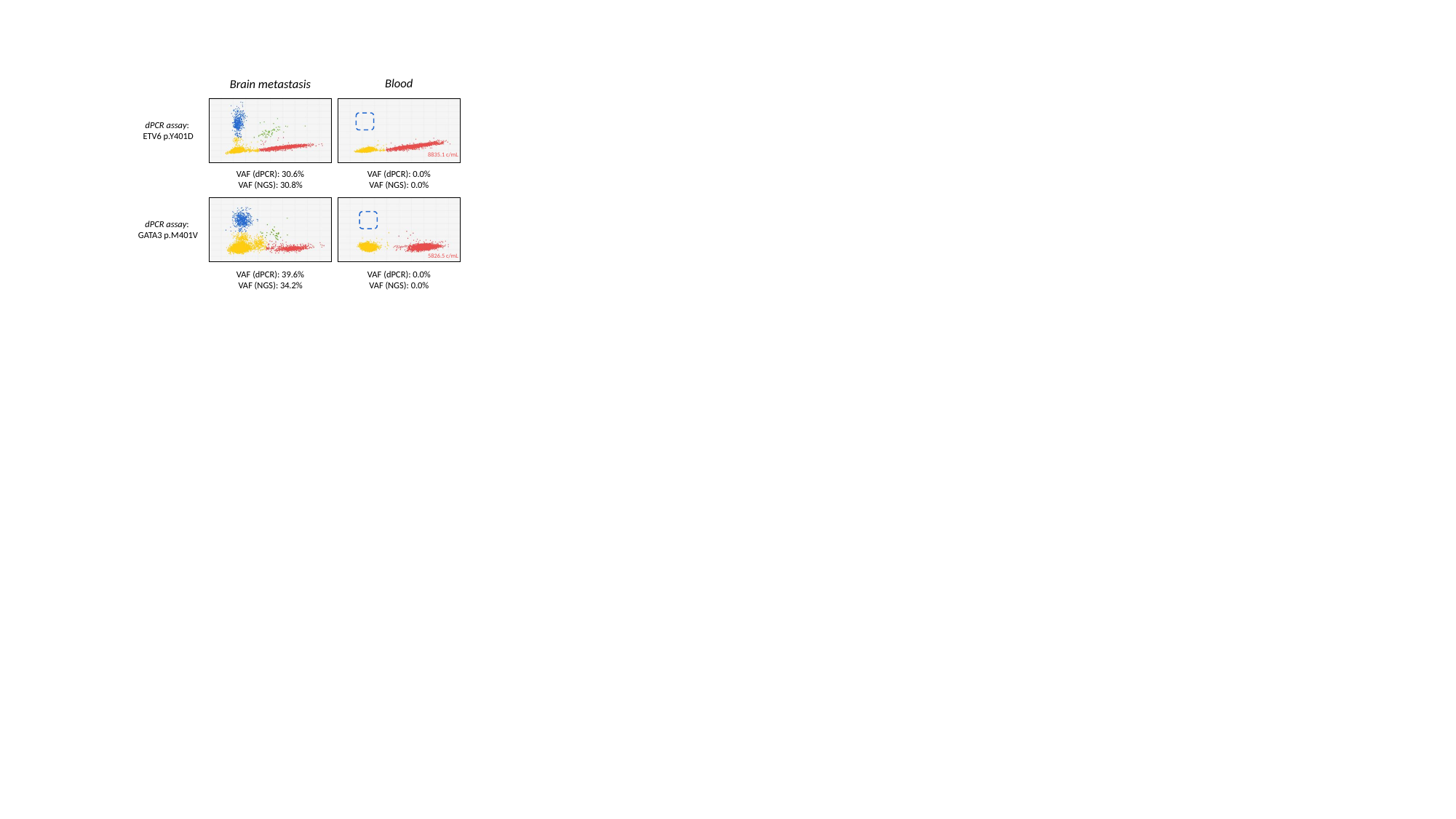

Blood
Brain metastasis
dPCR assay:
ETV6 p.Y401D
8835.1 c/mL
VAF (dPCR): 30.6%
VAF (NGS): 30.8%
VAF (dPCR): 0.0%
VAF (NGS): 0.0%
dPCR assay:
GATA3 p.M401V
5826.5 c/mL
VAF (dPCR): 39.6%
VAF (NGS): 34.2%
VAF (dPCR): 0.0%
VAF (NGS): 0.0%
