# Supplementary material for: Liquid biopsy identifies actionable dynamic predictors of resistance to Trastuzumab Emtansine (T-DM1) in advanced HER2-positive breast cancer
Source: Mol Cancer. 2021 Nov 29;20:151. doi: 10.1186/s12943-021-01438-z (PMC8628389; doi:10.1186/s12943-021-01438-z)
Supplement: Supplementary file 8 — Additional file 8: Table S1. Actionable level of circulating ctTNAs. * OncoKB highest level of evidence in advanced breast cancer. [file 12943_2021_1438_MOESM8_ESM.docx]

**Table S1. Actionable level of circulating ctTNAs. *** OncoKB highest level of evidence in advanced breast cancer.

| ***pt*** | ***ctTNA*** | ***Druggable*** | ***OncoKB level **** |
| --- | --- | --- | --- |
| #1 | TP53 p.R273H | N | - |
| #2 | PIK3CA p.H1047R | Y | 3A |
| #3 | ESR1 p.S463P | Y | 3A |
|  | ESR1 p.Y537C | Y | 3A |
| #4 | HER2 p.L755S | Y | 3A |
| #6 | PIK3CA p.E545K | Y | 3A |
| #7 | ESR1 p.Y537C | Y | 3A |
|  | ESR1 p.D538G | Y | 3A |
| #10 | PIK3CA p.H1047R | Y | 3A |
| #13 | HER2 p.V777L | Y | 3A |
| #17 | ESR1 p.D538G | Y | 3A |
|  | ESR1 p.Y537N | Y | 3A |
|  | ESR1 p.Y537S | Y | 3A |
|  | MYC amplification | N | - |
|  | FGFR1 amplification | Y | 4 |
| #18 | PIK3CA p.H1047R | Y | 3A |
|  | HER2 p.D769Y | Y | 3A |
| #19 | HER2 p.L755S | Y | 3A |
|  | PIK3CA p.G914R | Y | 3A |
| #20 | ESR1 p.S463P | Y | 3A |
| #21 | PIK3CA p.E545K | Y | 3A |
|  | TP53 p.G245S | N | - |
|  | MYC amplification | N | - |
| #22 | ESR1 p.D538G | Y | 3A |
