# Supplementary material for: Liquid biopsy identifies actionable dynamic predictors of resistance to Trastuzumab Emtansine (T-DM1) in advanced HER2-positive breast cancer
Source: Mol Cancer. 2021 Nov 29;20:151. doi: 10.1186/s12943-021-01438-z (PMC8628389; doi:10.1186/s12943-021-01438-z)
Supplement: Supplementary file 9 — Additional file 9. [file 12943_2021_1438_MOESM9_ESM.docx]

**Liquid biopsy identifies actionable dynamic predictors of resistance to Trastuzumab Emtansine (T-DM1) in advanced HER2-positive breast cancer**

Matteo Allegretti^1*^, PhD; Alessandra Fabi^2^*^, MD; Elena Giordani^1^; Cristiana Ercolani^3^; Paolo Romania^1^, PhD; Cecilia Nisticò^2^, MD; Simona Gasparro^2^, MD; Vittoria Barberi^4^, MD; Maria Ciolina^5^, MD; Edoardo Pescarmona^3^, MD; Diana Giannarelli^6^, PhD; Gennaro Ciliberto^7^, MD; Francesco Cognetti^8^, MD; Patrizio Giacomini^1^, MD.

^1^ Oncogenomics and Epigenetics, IRCSS Regina Elena National Cancer Institute, Rome, Italy

^2^ Medical Oncology 1, IRCSS Regina Elena National Cancer Institute, Rome, Italy

^3^ Pathology, IRCSS Regina Elena National Cancer Institute, Rome, Italy

^4^ Specialization School in Oncology, Sapienza University of Rome, Rome, Italy

^5^ Radiology and Diagnostic Imaging, IRCSS Regina Elena National Cancer Institute, Rome, Italy

^6^ Biostatistical Unit, IRCSS Regina Elena National Cancer Institute, Rome, Italy

^7^ Scientific Direction, IRCSS Regina Elena National Cancer Institute, Rome, Italy

^8^ Clinical and Molecular Medicine, Sapienza University of Rome, Rome, Italy

^^^ current affiliation: Precision Medicine Breast Cancer Unit, Fondazione Policlinico Universitario A. Gemelli IRCCS, Rome, Italy

* These authors equally contributed to the work

**Corresponding author**

Dr. Patrizio Giacomini

Oncogenomics and Epigenetics

IRCSS Regina Elena National Cancer Institute

Via Elio Chianesi, 53

00144, Rome, Italy

Phone: +39-0652662533

Fax: +39-0652662600

e-mail: [patrizio.giacomini@ifo.gov.it](mailto:patrizio.giacomini@ifo.gov.it)

**SUPPLEMENTARY METHODS**

**Patients and study design**

Eligible patients (n=22) had a histologically confirmed diagnosis of advanced HER2-positive breast cancer, an ECOG Performance Status ≤2, no concurrent malignant neoplasms, and a clinical and/or radiological documented progression following previous therapy lines, of which at least one comprising Trastuzumab with or without Pertuzumab plus taxane. Additional inclusion/exclusion criteria were as per standard of care (SoC). T-DM1 was administered at 3.6 mg/kg i.v. every 21 days until either progressive disease (evaluated as per clinical practice) or other events requiring discontinuation. Selected patients were monitored for 3 additional months after progression on T-DM1.

**Sample processing**

Sections (5 µm-thick) were cut from a representative formalin-fixed, paraffin-embedded (FFPE) tissue block or core biopsy from primary and/or metastatic lesions. One section was counterstained by hematoxylin/eosin and assessed for quality by an expert pathologist. The others were deparaffinized and digested overnight at 56°C with proteinase K (Qiagen, Hilden, Germany). Tissue DNA was extracted by the QIAmp DNA FFPE Tissue Kit (Qiagen) according to the manufacturer’s instructions. Whole blood (30ml) was drawn throughout the entire follow up of the patients on the occasion of T-DM1 administration in BD Vacutainer K_2_EDTA tubes and processed within 1h. Plasma was isolated by two successive rounds of centrifugation at 4°C (2000 x *g* for 20 min, and 13000 x *g* for 10 min), and stored at -80° in single-use 2 ml aliquots until circulating free total nucleic acids (cfTNAs) extraction. No freeze-thawing cycles were allowed. cfTNAs were extracted from 4ml of plasma by the QIAmp Circulating Nucleic Acid kit (Qiagen) according to the manufacturer’s instructions in a final volume of 30 µL, and stored at -20°C until analysis. Genomic DNA (gDNA) from matched plasma-depleted whole blood (e.g leukocytes) was extracted by the DNeasy Blood and Tissue kit (Qiagen) as indicated. Both tDNAs, gDNA and cfTNAs were fluorimetrically quantified with the Qubit dsDNA HS assay kit (Life Technologies, Carlsbad, CA, USA).

**Library preparation and sequencing**

Tissue NGS libraries were prepared from 10 ng of tDNA with the Ion AmpliSeq™ Library kit 2.0 and the Ion AmpliSeq™ Cancer Hotspot Panel v2 (Life Technologies), as per manufacturer’s instructions. This panel encompasses 2,800 COSMIC mutations from the 50 most commonly reported oncogenes and tumor suppressor genes. For blood NGS analysis, 40 ng of cfTNAs whenever available (mean 23.7 ng, range 4.4 – 40 ng), were used as input to prepare libraries with the Oncomine™ PanCancer Cell-Free Assay (Life Technologies) as per protocol. This panel generates an amplicon library from both ctDNA and ctRNA covering 52 genes, 12 copy number variations (CNVs) and 92 fusions. Libraries were equalized, pooled and then automatically loaded onto the Ion 520 (tissue) or 540 chips (blood) by the Ion Chef system (Life Technologies). After sequencing on Ion S5, data were analyzed with the Ion Reporter suite v5.16 (Life Technologies). LOD and sequencing depth for tissue/blood samples are reported in Fig S2. A custom filter chain including restriction on location (exonic), *p* value (<.05), variant effect (unknown, missense, nonsense, stoploss, frameshift insertions and deletions), variant type (SNV, small INDELS, MNV), filtered coverage (n=250) and VAF ≥2% was applied to filtering tissue NGS data. The preset Oncomine Variants filter v5.16 (Life Technologies) was used to call alterations in blood samples without any adjustments.

**Digital PCR**

Primers and probes for mutation calling were designed with the Custom Taqman® Assay Design Tool (CADT, Life Technologies). Matched tissue and blood samples from each patient and cfDNA from healthy donors were run in the same experiment using the chip-based QuantStudio™ 3D Digital PCR System (Life Technologies). Reactions were set up in a final volume of 16 μl including 8 μl of 2x Master Mix, 0.9 nM of each forward and reverse primers, 0.25 nM of TaqMan® MGB probe, 7.0 μl of template, and loaded onto dPCR chips. Input DNA for tissue analysis was normalized to 20 ng. By contrast, input cfDNA was equalized by volume (7.2 ul) to accurately measure ctDNA copies/mL of plasma. Thermal cycling was as follows: 10 min at 96.0°C, 39 cycles at 56.0°C for 2 min, 30 sec at 98.0°C, and a final elongation step of 2 min at 60°C. Threshold values of FAM and VIC fluorescence were automatically calculated by the Thermo Fisher Cloud Analysis Suite in tissue samples, manually reviewed, and then applied to the corresponding ctDNA.

**Assessment of the HER2 amplification status**

An existing, optimized dPCR assay was applied to calculate copy number ratios between the HER2 locus and the EFTUD2 reference gene(1-3). Unlike diagnostic CISH/FISH, this assay is applicable regardless of the analyte (tissue or blood) and is quantitative on a continuous scale, providing a unified readout. HER2:EFTUD2 thresholds of 2.0 and 1.25 were applied to define amplification in tissue and blood, as per HER2:CEP17 ASCOCAP guidelines(4) and original assay descriptions(1-3), respectively. HER2 amplification was also called as CNV by the Oncomine™ PanCancer Cell-Free Assay through the pre-set Oncomine Variant filter v 5.16 (Life Technologies).

**Statistical analysis**

Descriptive statistics were used to summarize patients and molecular features. Survival time was analyzed by the Kaplan-Meier method, and the log-rank test. The Wilcoxon signed-rank test was applied where indicated. Progression-free survival (PFS) was calculated as the time between the first T-DM1 administration and either the first evidence of progressive disease or the time of last follow-up. Variant allele frequencies (VAFs) in tumor tissue and blood samples were correlated by regression analysis. Two-sided *p* values <.05 were considered statistically significant. GraphPAD Prism v8.3 (GraphPad Software, CA, USA) was used for statistical and graphical elaborations.

**SUPPLEMENTARY REFERENCES**

1. Gevensleben H, Garcia-Murillas I, Graeser MK, Schiavon G, Osin P, Parton M, et al. Noninvasive detection of HER2 amplification with plasma DNA digital PCR. Clin Cancer Res. 2013;19(12):3276-84.

2. Page K, Hava N, Ward B, Brown J, Guttery DS, Ruangpratheep C, et al. Detection of HER2 amplification in circulating free DNA in patients with breast cancer. Br J Cancer. 2011;104(8):1342-8.

3. Garcia-Murillas I, Lambros M, Turner NC. Determination of HER2 amplification status on tumour DNA by digital PCR. PLoS One. 2013;8(12):e83409.

4. Wolff AC, Hammond MEH, Allison KH, Harvey BE, Mangu PB, Bartlett JMS, et al. Human Epidermal Growth Factor Receptor 2 Testing in Breast Cancer: American Society of Clinical Oncology/College of American Pathologists Clinical Practice Guideline Focused Update. J Clin Oncol. 2018;36(20):2105-22.
